# A High-Throughput Screening Approach To Repurpose FDA-Approved Drugs for Bactericidal Applications against Staphylococcus aureus Small-Colony Variants

**DOI:** 10.1128/mSphere.00422-18

**Published:** 2018-10-31

**Authors:** Ryan P. Trombetta, Paul M. Dunman, Edward M. Schwarz, Stephen L. Kates, Hani A. Awad

**Affiliations:** a Department of Biomedical Engineering, University of Rochester, Rochester, New York, USA; b Center for Musculoskeletal Research, University of Rochester Medical Center, Rochester, New York, USA; c Department of Microbiology and Immunology, University of Rochester Medical Center, Rochester, New York, USA; d Department of Orthopedics, University of Rochester Medical Center, Rochester, New York, USA; e Department of Orthopaedic Surgery, Virginia Commonwealth University School of Medicine, Richmond, Virginia, USA; University of Nebraska Medical Center

**Keywords:** *Staphylococcus aureus*, small-colony variants, high-throughput screen, drug repurposing, chronic infection

## Abstract

Drug repurposing offers an expedited and economical route to develop new clinical therapeutics in comparison to traditional drug development. Growth-based high-throughput screening is concomitant with drug repurposing and enables rapid identification of new therapeutic uses for investigated drugs; however, this traditional method is not compatible with microorganisms with abnormal growth patterns such as Staphylococcus aureus small-colony variants (SCV). SCV subpopulations are auxotrophic for key compounds in biosynthetic pathways, which result in low growth rate. SCV formation is also associated with reduced antibiotic susceptibility, and the SCV’s ability to revert to the normal cell growth state is thought to contribute to recurrence of S. aureus infections. Thus, there is a critical need to identify antimicrobial agents that are potent against SCV in order to effectively treat chronic infections. Accordingly, here we describe adapting an adenylate kinase (AK)-based cell death reporter assay to identify members of a Food and Drug Administration (FDA)-approved drug library that display bactericidal activity against S. aureus SCV. Four library members, daunorubicin, ketoconazole, rifapentine, and sitafloxacin, exhibited potent SCV bactericidal activity against a stable S. aureus SCV. Further investigation showed that sitafloxacin was potent against methicillin-susceptible and -resistant S. aureus, as well as S. aureus within an established biofilm. Taken together, these results demonstrate the ability to use the AK assay to screen small-molecule libraries for SCV bactericidal agents and highlight the therapeutic potential of sitafloxacin to be repurposed to treat chronic S. aureus infections associated with SCV and/or biofilm growth states.

**IMPORTANCE** Conventional antibiotics fail to successfully treat chronic osteomyelitis, endocarditis, and device-related and airway infections. These recurring infections are associated with the emergence of SCV, which are recalcitrant to conventional antibiotics. Studies have investigated antibiotic therapies to treat SCV-related infections but have had little success, emphasizing the need to identify novel antimicrobial drugs. However, drug discovery is a costly and time-consuming process. An alternative strategy is drug repurposing, which could identify FDA-approved and well-characterized drugs that could have off-label utility in treating SCV. In this study, we adapted a high-throughput AK-based assay to identify 4 FDA-approved drugs, daunorubicin, ketoconazole, rifapentine, and sitafloxacin, which display antimicrobial activity against S. aureus SCV, suggesting an avenue for drug repurposing in order to effectively treat SCV-related infections. Additionally, this screening paradigm can easily be adapted for other drug/chemical libraries to identify compounds bactericidal against SCV.

## Abstract (precis)

Conventional antibiotics fail to successfully treat chronic osteomyelitis, endocarditis, and device-related and airway infections. These recurring infections are associated with the emergence of SCV, which are recalcitrant to conventional antibiotics. Studies have investigated antibiotic therapies to treat SCV-related infections but have had little success, emphasizing the need to identify novel antimicrobial drugs. However, drug discovery is a costly and time-consuming process. An alternative strategy is drug repurposing, which could identify FDA-approved and well-characterized drugs that could have off-label utility in treating SCV. In this study, we adapted a high-throughput AK-based assay to identify 4 FDA-approved drugs, daunorubicin, ketoconazole, rifapentine, and sitafloxacin, which display antimicrobial activity against S. aureus SCV, suggesting an avenue for drug repurposing in order to effectively treat SCV-related infections. Additionally, this screening paradigm can easily be adapted for other drug/chemical libraries to identify compounds bactericidal against SCV.

## INTRODUCTION

Drug discovery is a crucial process to identify candidate compounds, molecules, and biologics that can potentially be developed into clinically effective therapeutics. However, this discovery process is costly and fraught with risk of frustrating failures. The associated costs of a successful drug launch can reach $800 million in research and development expenses, and the drug can take up to 15 years to develop (1, 2). Despite these huge expenditures, nearly 86.2% of drug candidates that make it to phase 1 trials fail to achieve drug approval (3). The time, costs, and high failure rate of drug development have prompted pharmaceutical companies to pursue alternative avenues for effective therapeutics. One promising avenue is drug repositioning or repurposing, which is the process of discovering new uses for existing drugs. Drugs that are Food and Drug Administration (FDA) approved and repurposed have the ability to go directly to preclinical and clinical trials, reducing the lengthy amount of time for preclinical drug development and, thus, reducing costly risks of failure (4). There are numerous examples of success using this repurposing approach, including screens that have identified candidate therapies for the ZIKV infection, Ebola virus disease, Alzheimer’s disease, and hepatitis C virus (5–8).

A commonly applied method to identify off-label effects of FDA-approved drugs for drug repurposing is to perform high-throughput screens (HTS) of drug compound libraries (9–13). This methodology is especially critical in the context of chronic bacterial infections due to the rising bacterial resistance to antibiotics (14). Drug-resistant bacterial strains can be easily screened using whole-cell, bacterial growth assays against drug and compound libraries. However, in addition to drug resistance, chronic infections are often attributed to alternative bacterial phenotypes such as small-colony variants (SCV). SCV are subpopulations of bacteria with slower metabolism and are thought to be a key contributor to chronic Staphylococcus aureus infections. The altered growth state of SCV is characterized by a low growth rate, reduced coagulase and hemolytic activity, small colony size on agar plates, absence of pigmentation, antibiotic recalcitrance, and varied auxotrophy for hemin, menadione, and/or thymidine, which is required for normal growth (15–17). Because of their abnormal and low growth rate, conventional whole-cell, growth-based assays used for HTS are not practical with SCV, urging the need for other HTS methodologies.

SCV are quite common from an epidemiological standpoint, and there is a growing appreciation of the role the reduced growth state plays in chronic infections such as osteomyelitis, endocarditis, device infections, soft tissue infections, and airway infections (15, 18–27). For instance, staphylococcal SCV have been recovered in 34% of prosthetic joint infections (PJI) and between 8 and 63.5% of cystic fibrosis cases (16, 18–21). SCV identification and diagnosis are particularly difficult, and SCV are often overlooked due to their reduced growth rate and small colony size, as well as lack of pigmentation and reduced coagulase activity, leading to misidentification as coagulase-negative staphylococci (16). Not all recovered SCV are physiologically the same, and they display phenotypic variation from patient to patient (21, 28). Moreover, while β-lactams, aminoglycosides, clindamycin, and fluoroquinolones are frontline therapies for the treatment of S. aureus infections, SCV are recalcitrant to these drugs due to their altered metabolism, prompting numerous studies to define the genetic determinants involved (29).

The reduced susceptibility of SCV to antibiotics is associated with their auxotrophism to menadione, hemin, and/or thymidine, resulting in deficiencies in electron transport (menadione and hemin) and/or the thymidylate biosynthetic pathway (30). The deficiencies in these metabolic pathways result in lower membrane potential and reduced metabolism, which explain SCV’s ability to survive antibiotic treatment (31, 32). Additionally, SCV have the ability to persist intracellularly within nonphagocytic cells such as bone, epithelial, and endothelial cells, hence avoiding host-immune response and antibiotic treatment (33, 34). The ability of SCV to revert to normal colony phenotype (NCP) also adds evidence for their important role in the reoccurrence of infection and the restoration of virulence, leading to recurrent infection. Thus, there is a clinical need to prioritize current antibiotics, reposition drugs, and/or identify novel agents that are effective against SCV to prevent relapses in infection and to effectively treat chronic infections.

The objective of this study was to perform a high-throughput screen (HTS) of an FDA-approved drug library to specifically identify candidates that are not only bactericidal against NCP and biofilm but also bactericidal to a mutated small-colony variant strain (SCV) harboring a *hemB* deletion, UAMS-1112 (Δ*hemB*). With this objective in mind, we set out to adapt a recently developed and validated adenylate kinase (AK) release reporter assay as an HTS platform to identify agents that display bactericidal activity toward slowly and/or nongrowing bacterial populations, including SCV (35). This HTS has recently been used to screen an FDA-approved drug library for agents that displayed bactericidal activity against *Pseudomonas aeruginosa* biofilms, identifying 34 compounds that displayed antibiofilm activity (36). The basis for the assay is that AK is a ubiquitous intracellular enzyme that catalyzes the conversion of two ADP molecules into ATP and AMP, which is released extracellularly after cell death. Extracellular AK release from lysed cells can subsequently be measured using commercial ToxiLight reporter cocktail based on the ATP-dependent bioluminescent measurement of AK (Lonza, Basel, Switzerland). In this study, we demonstrate that this AK release assay can be scaled for HTS of a Food and Drug Administration (FDA)-approved drug library containing 853 drug candidates and that it can identify candidates that are bactericidal against UAMS-1112 (Δ*hemB*). Assay hits were then further validated and characterized for their antimicrobial efficacy against wild-type S. aureus strains, antibiofilm activity, resistance frequency, and human cell cytotoxicity.

## RESULTS

### Identification of screening hits that are bactericidal against S. aureus small-colony variants.

The AK-based HTS assay was used to screen a Selleck library consisting of 853 FDA-approved drug candidates that showed bactericidal activity at 100 μM after 24 h of treatment against a stable SCV S. aureus strain, UAMS-1112 (Δ*hemB*) (see Table S1 in the supplemental material). Screening identified 22 drugs that displayed an increase in AK signal that surpassed two standard deviations above the overall mean AK level for the entire library (Table 1). Among these, two antibiotics, rifapentine (an antitubercular agent) and sitafloxacin (a fluoroquinolone) were identified. The remaining 20 varied in therapeutic area classifications ranging from antifungal (ketoconazole), anticancer (10-DAB, apatinib, daunorubicin), antiviral (ammonium glycyrrhizinate, cidofovir), cardiac related (clonidine hydrochloride, furosemide, milrinone), β-lactamase inhibitor (sulbactam), proton pump inhibition (esomeprazole), antioxidant/anthelmintic (genistein), anesthetic (mepivacaine), muscle relaxant (methocarbamol), antihistamine (mizolastine), antiangiogenic/immunomodulator (pomalidomide), water-soluble vitamin (pantothenic acid), sulfonamide (sulfamethazine), to antipsychotic/antidepressant (amitriptyline, olanzapine).

**TABLE 1:** Selleck library small-colony variant (SCV) screening hits

Drug	Avg fold change in AK signal (SD)^*a*^
10-DAB (10-deacetylbaccatin)	1.52 (±0.65)
Amitriptyline	1.44 (±0.49)
Ammonium glycyrrhizinate (AMGZ)	1.58 (±0.86)
Apatinib	1.71 (±1.03)
Cidofovir	1.17 (±0.03)
Clonidine hydrochloride	1.66 (±1.02)
Daunorubicin	1.44 (±0.30)
Esomeprazole	1.78 (±1.06)
Furosemide	1.24 (±0.25)
Genistein	1.32 (±0.08)
Ketoconazole	1.30 (±0.07)
Mepivacaine	1.45 (±0.57)
Methocarbamol	1.24 (±0.05)
Milrinone (Primacor)	1.62 (±0.86)
Mizolastine (Mizollen)	1.59 (±0.95)
Olanzapine (Zyprexa)	1.50 (±0.67)
Pantothenic acid	1.73 (±0.99)
Pomalidomide	1.20 (±0.15)
Rifapentine	1.18 (±0.08)
Sitafloxacin	1.42 (±0.58)
Sulbactam	1.26 (±0.36)
Sulfamethazine	1.47 (±0.51)

a Data represent average fold increase in adenylate kinase (AK) release in comparison to mock-treated control.

10.1128/mSphere.00422-18.1 TABLE S1 Drug list of Selleck library. Download Table S1, XLSX file, 0.03 MB. Copyright © 2018 Trombetta et al. 2018 Trombetta et al. This content is distributed under the terms of the Creative Commons Attribution 4.0 International license.

**FIG 1:**
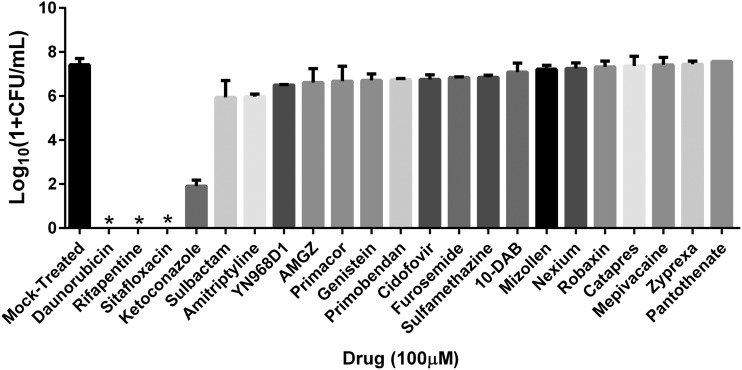
Validation of the 22 screening hits against S. aureus small-colony variants (SCV). The 22 identified potential drug candidates that exhibited an adenylate kinase (AK) signal that surpassed hit selection criteria were assessed for antimicrobial efficacy against SCV at 100 μM. Of the 22 hits, 4 drugs, daunorubicin, ketoconazole, rifapentine, and sitafloxacin, demonstrated a ≥5-log reduction of viable bacteria in comparison to a mock-treated control, such that daunorubicin, rifapentine, and ketoconazole came back culture negative. Assay was performed in duplicate. * indicates culture-negative. Data presented as mean ± standard deviation.

To confirm whether the increased AK signal did indeed correspond to bactericidal activity of the drugs, each of the 22 screening hits was retested for antimicrobial activity using quantitative microbiology by plating UAMS-1112 (Δ*hemB*) and treating with each candidate drug at 100 µM. For each drug, the resulting number of viable CFU were compared to mock-treated cells (dimethyl sulfoxide [DMSO]). Four of the 22 drugs tested, daunorubicin, ketoconazole, rifapentine, and sitafloxacin, displayed potent antimicrobial activity resulting in ≥5-log reduction in viable SCV compared to mock-treated cells. Of these, daunorubicin, an anthracycline chemotherapy agent; rifapentine, a rifamycin class antibiotic; and sitafloxacin, a fluoroquinolone antibiotic, yielded no detectable viable bacteria while ketoconazole, an imidazole antifungal, displayed a 5.52-log reduction relative to the mock-treated control (Fig. 1). The remaining 18 hits displayed ≤1.5-log reduction and, thus, were considered less potent and classified as low-priority agents that were not investigated further.

### MICs of validated screening hits against S. aureus SCV and NCP strains.

As an initial means to evaluate the spectrum of antimicrobial activity of the four high-priority hits, conventional MIC assays were performed on genetically diverse strains of S. aureus including the methicillin-susceptible S. aureus (MSSA) strain UAMS-1, methicillin-resistant S. aureus (MRSA) strain USA300, and UAMS-1112 (SCV; Δ*hemB*) for daunorubicin, ketoconazole, rifapentine, and sitafloxacin at concentrations ranging from 0 to 128 μg/ml. MIC results were compared to that of a conventional aminoglycoside, gentamicin (Table 2), which is commonly used in local delivery for orthopedic infections via poly(methyl methacrylate) (PMMA) bone cement spacers (37, 38). The observed 8-fold increase in MIC of gentamicin against SCV in comparison to the NCP UAMS-1 was consistent with previously reported values and reaffirmed that SCV are refractory to the antibiotic (39, 40). Rifapentine and sitafloxacin had MICs against SCV and the other strains tested that were lower than gentamicin, daunorubicin, and ketoconazole (Table 2), suggesting they may represent potent options for treating S. aureus SCV-associated infections.

**TABLE 2:** Antimicrobial susceptibility of 4 validated drugs and gentamicin

Drug	MIC (µg/ml)			
	UAMS-1	USA300	UAMS-1112 (SCV; Δ*hemB*)	UAMS-1 serum^*a*^
Daunorubicin	8	8	4	8
Ketoconazole	32	32	16	>128
Rifapentine	0.016	0.032	0.032	1
Sitafloxacin	0.016	0.125	0.032	0.25
Gentamicin	0.5	1	4	2

a Susceptibility testing performed in human serum. All other testing performed in Mueller-Hinton (MH) broth.

### Antimicrobial efficacy of validated screening hits against established S. aureus biofilm.

**FIG 2:**
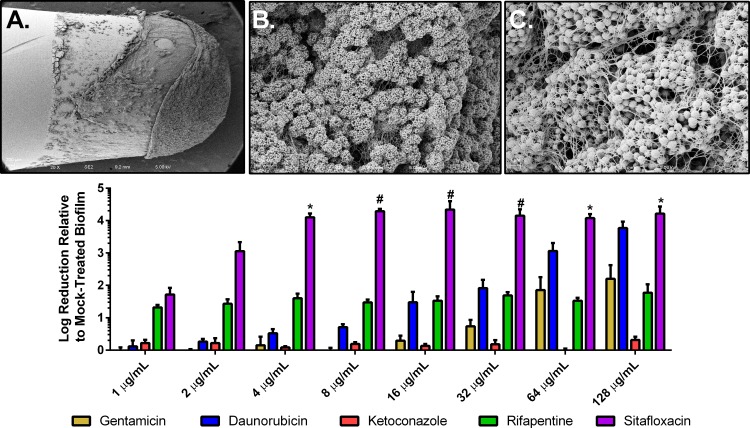
Dose response of gentamicin, daunorubicin, ketoconazole, rifapentine, and sitafloxacin against established S. aureus biofilm using the MBEC assay. UAMS-1 biofilm was established on polystyrene pegs after 24 h of incubation with media supplemented with 10% (vol/vol) human plasma, and representative SEM is shown with magnifications of ×100 (A), ×1,000 (B), and ×5,000 (C). Biofilm was challenged by 1 to 128 µg/ml of each of the 4 validated HTS drugs and gentamicin (D). Sitafloxacin significantly killed biofilm at concentrations 4 to 128 µg/ml in comparison to gentamicin at each concentration. * indicates *P* < 0.005; # indicates *P* < 0.001 for the effect of gentamicin versus daunorubicin, ketoconazole, rifapentine, or sitafloxacin at each of the respective concentrations by 2-way ANOVA with Sidak’s test for *post hoc* comparisons. *n* = 3/group. Data presented as mean ± standard deviation.

S. aureus chronic infections are also associated with biofilm formation, which is comprised of a heterogenous population of cells, both NCP and SCV (41, 42). To further assess the antimicrobial efficacy of the identified candidates, S. aureus biofilms were grown for 24 h and then challenged with daunorubicin, ketoconazole, sitafloxacin, rifapentine, and gentamicin to assess biofilm vulnerability to these compounds. Briefly, UAMS-1 biofilms were grown on polystyrene pegs using the minimum biofilm eradication concentration (MBEC) assay (Innovotech, Edmonton, Canada). Growth medium was supplemented with 10% (vol/vol) human plasma (43), which enabled robust biofilm to be formed after 24 h (Fig. 2A to C). Established biofilms were then challenged with daunorubicin, ketoconazole, sitafloxacin, rifapentine, or gentamicin for 24 h at concentrations ranging from 1 to 128 µg/ml. Pegs were then sonicated in recovery plates, serially diluted, and then plated, and resulting CFU were enumerated. Sitafloxacin demonstrated a ≥4-log reduction in recovered biofilm-associated CFU in comparison to the mock-treated growth control (DMSO) at concentrations ≥4 µg/ml (Fig. 2D). Additionally, compared to gentamicin treatment (control), sitafloxacin displayed significant reduction of viable bacteria within biofilms at these concentrations. Gentamicin and rifapentine induced no more than a 2-fold reduction in recovered biofilm-associated CFU at all concentrations tested. Daunorubicin resulted in ∼4-log reduction in recovered biofilm-associated CFU only at concentrations >64 µg/ml. Ketoconazole failed to demonstrate any significant bactericidal activity against S. aureus biofilm over the entire range of concentrations tested.

### Mammalian cytotoxicity of validated screening hits.

**FIG 3:**
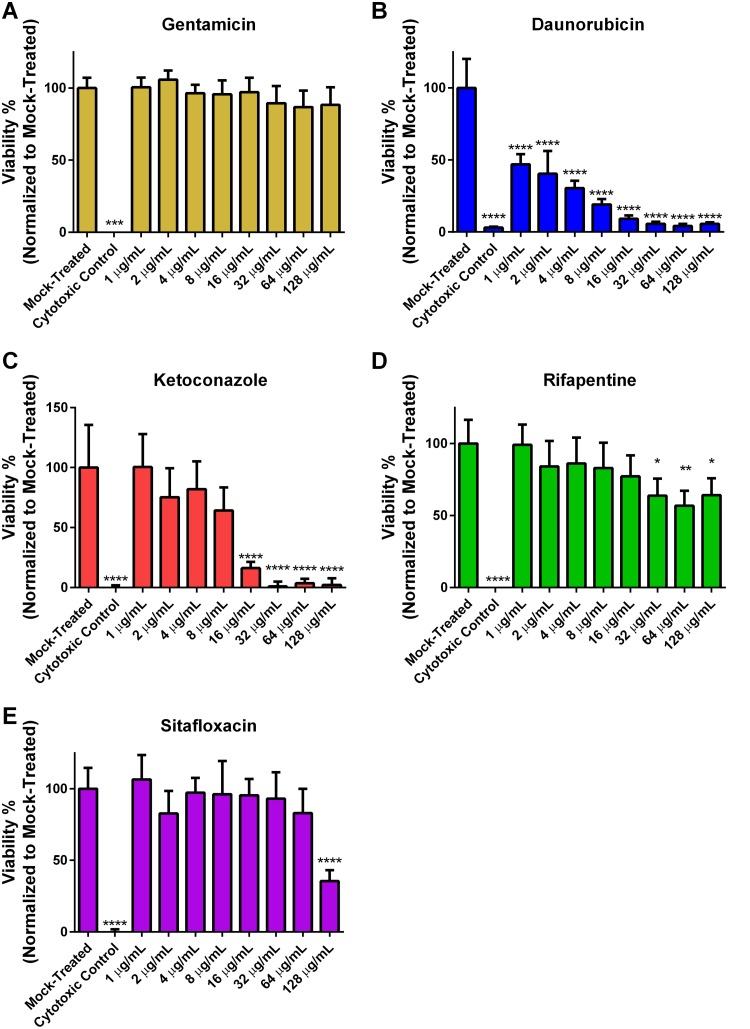
Cytotoxicity of gentamicin, daunorubicin, ketoconazole, rifapentine, and sitafloxacin against human embryonic kidney (HEK) 293T cells. Gentamicin and each of the 4 validated screening hits were screened for cytotoxic effects at concentrations 1 to 128 mg/ml against HEK 293T cells using an XTT proliferation assay. Significance was judged for the effect of each drug concentration versus the mock-treated control by 2-way ANOVA with Sidak’s test for *post hoc* comparisons. * indicates *P* < 0.05, ** indicates *P* < 0.01, *** indicates *P* < 0.005, and **** indicates *P* < 0.001. *n* = 3/group. Data presented as mean ± standard deviation. Negative control was 64 mg/ml of zinc pyrithione.

To assess the detrimental effects of the drug candidates on mammalian cells and to establish a therapeutic window by comparing toxic concentrations with identified MICs, the cytotoxicity of daunorubicin, ketoconazole, rifapentine, sitafloxacin, and gentamicin was measured using an XTT proliferation assay, which involved treating human embryonic kidney (HEK) 293T cells with the candidate drugs at concentrations ranging from 0 to 128 μg/ml for 24 h. Results showed that gentamicin did not induce significant cytotoxic effects over the range of concentrations tested, with >85% cell viability (Fig. 3A). Sitafloxacin and rifapentine displayed the least cytotoxic effects of the candidates tested, with no significant dose-dependent cytotoxicity effects observed at concentrations ranging from 1 to 64 μg/ml and 1 to 16 μg/ml, respectively (Fig. 3D and E), whereas daunorubicin and ketoconazole demonstrated significant cytotoxic effects in comparison to mock-treated cells at all concentrations ranging from 1 to 128 μg/ml and 16 to 128 μg/ml, respectively (Fig. 3B and C).

The therapeutic ratio, defined as the ratio of the lowest concentration causing cytotoxicity to the MIC of each tested S. aureus strain, was calculated for daunorubicin, ketoconazole, rifapentine, and sitafloxacin (Table 3). Consistent with the above observations, daunorubicin and ketoconazole had the lowest therapeutic ratios (<0.25 and <1, respectively), whereas rifapentine and sitafloxacin had the highest therapeutic ratios (>1,000).

**TABLE 3:** Therapeutic ratios of validated screening hits

Drug	UAMS-1	USA300	Small-colony variants
Daunorubicin	<0.125	<0.125	<0.25
Ketoconazole	0.5	0.5	1
Rifapentine	2,000	1,000	1,000
Sitafloxacin	8,000	1,024	4,000

### Antimicrobial activity in human serum and spontaneous resistance frequency.

The antimicrobial activity against UAMS-1 in human serum was also evaluated to investigate the bioactivity of each drug in the presence of serum proteins. The MIC was generally increased, reflecting >4-, 62.5-, 15.63-, and 4-fold decreases in bactericidal activity for ketoconazole, rifapentine, sitafloxacin, and gentamicin in the presence of human serum, respectively (Table 1). However, no change was observed for daunorubicin in the presence of human serum, indicating that serum proteins do not affect its bactericidal activity. Although sitafloxacin demonstrated serum inactivation liabilities (∼16-fold increase in MIC), the MIC in serum was 0.25 μg/ml, still below that of the clinical control gentamicin’s MIC of 0.5 μg/ml in regular Mueller-Hinton medium, and well within the therapeutic window demonstrating its bactericidal potency and safety in either setting.

The spontaneous resistance frequencies were also measured at 2× and 4× MIC for daunorubicin, ketoconazole, rifapentine, and sitafloxacin, as well as rifampin, to investigate the development of resistant mutants when UAMS-1 was subjected to each drug. Rifampin was used as a control because it is a known antibiotic that readily induces S. aureus resistant mutants (44). Ketoconazole had the highest frequency of resistance on the order of 10^−5^ at either 2× or 4× MIC (Table 4). Rifapentine and rifampin all had similar spontaneous resistance frequencies on the order of 10^−7^ at 2× or 4× MIC. However, while sitafloxacin had similar equivalent resistance frequencies to rifapentine and rifampin at 4× MIC, it demonstrated an order of magnitude higher resistance frequency at 2× MIC. Daunorubicin yielded no detectable levels of resistant mutants at 2× or 4× MIC. These data suggest that rifapentine and sitafloxacin are equivalent to rifampin in their induction of resistant mutants, while ketoconazole was the most likely to produce resistant mutants. Monotherapy of these drugs will result in the incidence of resistant mutants; however, the addition of a supplemental drug, such as vancomycin, as combination therapy can reduce resistance.

**TABLE 4:** Spontaneous resistance frequencies of validated screening hits and rifampin against UAMS-1

Drug	Spontaneous resistance frequency (SD)	
	2× MIC	4× MIC
Daunorubicin	<1.12 × 10^−9^ (±1.65 × 10^−8^)	<1.17 × 10^−9^ (±2.33 × 10^−10^)
Ketoconazole	1.65 × 10^−5^ (±2.12 × 10^−6^)	5.00 × 10^−5^ (±1.70 × 10^−5^)
Rifapentine	4.74 × 10^−7^ (±3.38 × 10^−7^)	1.23 × 10^−7^ (±3.54 × 10^−9^)
Sitafloxacin	5.68 × 10^−6^ (±3.44 × 10^−6^)	2.5 × 10^−7^ (±1.04 × 10^−7^)
Rifampin	4.40 × 10^−7^ (±2.76 × 10^−7^)	1.71 × 10^−7^ (±1.56 × 10^−9^)

## DISCUSSION

S. aureus SCV are a clinical challenge due to their association with recurrent and antibiotic-resistant infections such as implant-associated osteomyelitis (18). Conventional therapeutic treatments utilize antibiotics, such as aminoglycosides, that have decreased potency against SCV (15, 19, 25, 40). For example, localized delivery of antibiotics via PMMA bone cement spacers laden with gentamicin fails to eradicate SCV and is not effective in preventing reoccurrence of infection in cases of osteomyelitis (15). Due to their reduced susceptibility to conventional antibiotics, novel drug discovery and development are essential for efficacious treatments against SCV-related chronic infections. However, drug discovery is a notoriously slow, expensive, and high-risk process. Poor profitability and uncertain regulatory requirements for market approval have slowed discovery initiatives from large pharmaceutical companies, which led to a critical need to find new approaches for new antimicrobial drug development (45–47). One such approach is drug repurposing, which enables an expedited and efficient strategy for identifying new applications for approved drugs to treat disease (48). Incidentally, 30% of newly FDA-approved drugs are repurposed agents (49). Conventional high-throughput screens typically use whole-cell, bacterial growth assays, which makes screening very challenging due to the slow and abnormal growth characteristics of SCV. Hence, this study uses an AK release-based cell death reporter assay (35) for an HTS of an FDA-approved Selleck drug library for bactericidal activity against S. aureus SCV. We demonstrate the ability of this screening approach to identify 4 FDA-approved drugs, daunorubicin, ketoconazole, rifapentine, and sitafloxacin, as bactericidal drugs against S. aureus SCV.

Current efforts aim to identify antibiotics effective against SCV by investigating the susceptibility of stable clinical isolates or site-directed SCV mutants *in vitro* using conventional MIC testing or using animal models of infection (40). In general, these studies have consistently shown that antibiotics, including aminoglycosides, cationic compounds, and antifolates, have less activity against SCV due to their lower membrane potential relative to NCP (15, 50, 51). Additionally, cell-wall-active antibiotics and antibiotics that inhibit protein biosynthesis have been shown to have reduced bactericidal activity against the slow-growing SCV (52). Yet, some antibiotics belonging to the families of rifamycins and fluoroquinolones have shown effectiveness against SCV *in vitro,* demonstrating a low MIC ranging from 0.0005 to 0.06 μg/ml and 0.06 to 0.5 μg/ml, respectively (25, 40, 53). Furthermore, rifamycins have shown efficacy in clearing intracellular SCV, as well as reducing the bacterial burden during the acute phase in a long-term osteomyelitis murine mode (25). The potency of rifamycins and fluoroquinolones is consistent with the results of our HTS, which identified rifapentine, a rifamycin, and sitafloxacin, a fluoroquinolone, as bactericidal against a stable SCV *hemB* mutant. Although rifapentine and sitafloxacin belong to two classes of antibiotics that have generally shown some promise against SCV, this is the first time these two specific drugs have been identified to directly kill SCV. Additionally, screening the Selleck library investigated not only FDA-approved antibiotics, but a broad spectrum of FDA-approved drugs developed for other therapeutic applications. This enabled us to identify two nonantibiotic drugs that were bactericidal against S. aureus SCV, daunorubicin and ketoconazole. Incidentally, daunorubicin, an anthracycline chemotherapy agent, and ketoconazole, an imidazole antifungal, have previously been shown to have a bactericidal mode of action against S. aureus (54, 55). However, this is the first study to confirm their bactericidal effectiveness against S. aureus SCV.

Daunorubicin, ketoconazole, rifapentine, and sitafloxacin had varied MICs against S. aureus SCV and NCP regardless of drug mode of action. Both sitafloxacin and daunorubicin inhibit DNA synthesis by acting upon type II DNA topoisomerases (56, 57); however, the MIC of sitafloxacin was found to be nearly two orders of magnitude lower than daunorubicin. These differences may be attributed to the uptake of drug by the SCV phenotype, as well as sitafloxacin’s additional mode of action against DNA gyrase, warranting further investigation of sitafloxacin’s efficacy against SCV (57). Rifapentine had a similar SCV MIC of 0.032 μg/ml in regard to sitafloxacin even though its mode of action inhibits the β-subunit of the bacterial RNA polymerase (58). Ketoconazole had the highest MIC, which is consistent with its inhibition of sterol synthesis, and is most effective in antifungal therapies. However, in regard to S. aureus, imidazole antifungals induce intracellular reactive oxygen species production (54). Yet, despite the different modes of action, no synergy between any of the 4 identified drugs was observed by fractional inhibitory concentration (data not shown).

Further drug characterization was performed to assess the efficacy of each of these four drugs against various S. aureus strains and biofilm and cytotoxicity. The results of this characterization demonstrated sitafloxacin was the best-performing drug due to its low MIC against SCV and various S. aureus strains, its potency against established biofilm, and its low cytotoxicity. However, the antibiotic had a comparable spontaneous resistance frequency to that of rifampin, which is known to produce resistant mutants in clinic when treated as a monotherapy, suggesting the need to be used in combination therapy to mitigate this issue (61, 62). Sitafloxacin was developed in Japan and approved in 2008 for treating respiratory and urinary tract infections (63). Here, we showed sitafloxacin’s efficacy against SCV and biofilm, which has not been reported (59). This is significant since implant-associated osteomyelitis is a chronic infection associated with both SCV and biofilm. Thus, we propose that a repurposing approach using the AK release assay as the basis of HTS can be an effective approach to identify new uses of approved drugs for the treatment of infections, including SCV. For example, the repurposing of sitafloxacin for this orthopedic infection could prove valuable for its potential efficacy in the treatment of implant-associated osteomyelitis. This approach has decided advantages in saving money and time as opposed to novel drug development (60). The full potential of this assay as an HTS tool is that it can be scalable to large drug or novel chemical libraries, which should be screened not only against lab strains but also against various clinical isolates of SCV strains associated with many chronic infections.

The results of this study serve as the foundation for developing more potent therapies for combating recurrent infections. The high therapeutic ratios of sitafloxacin and rifapentine could serve as the basis for refining the pharmacophore structure of these drugs to increase their bactericidal potency and to further extend the screening paradigm to interrogate novel small-molecule drug candidates to develop new classes of antimicrobials to effectively combat SCV in recurrent infections. However, this study has limitations. One caveat of using the AK assay to screen against SCV is that we found that 18 of the 22 identified hits had a ≤1-log reduction against SCV, resulting in an 82% false-positive hit rate. This rate could be decreased by refining the selection criteria for positive hits; however, this would come at the risk of missing positive hit identification. Additionally, identified hits were those associated with lytic cell death, while compounds associated with nonlytic cell death would have been missed. A further limitation to this study is that only one stable SCV mutant (Δ*hemB*) was used for screening purposes. Clinically recovered SCV are a heterogenous population varying in characteristics and antibiotic susceptibility based on their auxotrophism for distinct growth factors (51, 64). The resulting metabolic pathway deficiencies affect antibiotic susceptibility of recovered SCV. Therefore, future work will investigate the potency of each of the identified hits against other stable SCV mutants (i.e., Δ*menD*) as well as clinical isolates.

In summary, we utilized an AK release cytotoxicity assay-based HTS and identified 4 drugs, daunorubicin, ketoconazole, rifapentine, and sitafloxacin, that display bactericidal potency against a stable Δ*hemB* SCV strain (UAMS-1112). Identification of these drugs demonstrates the usefulness of this approach in identifying bactericidal agents against SCV and provides a strategy for drug repurposing to treat chronic infections associated with S. aureus SCV. Among these drugs, sitafloxacin displayed the highest potential in treating such infections due to its high therapeutic ratio and its significant effectiveness against S. aureus biofilm in comparison to gentamicin, which is the clinical standard for treating osteomyelitis. Future studies will continue to screen drug and chemical libraries using the AK screen against SCV but also investigate the efficacy and safety of sitafloxacin in combination with clinically used antibiotics in the treatment of established, implant-associated osteomyelitis in a preclinical rodent model (65).

## MATERIALS AND METHODS

### Bacterial strains and growth conditions.

The bacterial strains used in this study consist of S. aureus strain UAMS-1112, a stable SCV of the common laboratory S. aureus strain 8325-4, which contains a *hemB* deletion (a kind gift from M. Smeltzer, University of Arkansas Medical Center, Little Rock, AR), UAMS-1, a methicillin-susceptible S. aureus (MSSA) clinical isolate, and USA 300, a community-associated, methicillin-resistant strain of S. aureus (MRSA). Overnight cultures of UAMS-112 were grown for 24 to 36 h in Mueller-Hinton (MH) medium supplemented with 10 µg/ml of erythromycin at 37°C on a rotary shaker at 225 rotations per min (rpm). All other overnight cultures were grown for 16 h in either Mueller-Hinton (MH) medium or tryptic soy broth (TSB).

### Chemicals and compound libraries.

A library consisting of 853 FDA-approved drugs was purchased from Selleck Chemical (Houston, TX). Daunorubicin, sitafloxacin, and rifapentine were also obtained from Selleck Chemical. Ketoconazole and gentamicin sulfate were purchased from Alfa-Aesar (Ward Hill, MA). Antimicrobial stocks were prepared according to recommendations and stored at −20°C.

### Adenylate kinase bactericidal reporter assay.

An 853-member FDA-approved drug library was screened against SCV using an AK HTS. AK assays were carried out as previously described with modifications to account for the slower growth of UAMS-1112 (35, 36). Briefly, 36-h cultures of the stable S. aureus SCV strain, UAMS-1112 (Δ*hemB*), were grown in MH medium at 37°C at 225 rpm, used to inoculate (1:100 dilution) 500 ml of MH medium, and grown to an optical density (OD_600_) of 0.2 corresponding to ∼1 × 10^6^ CFU/ml. Cells were pelleted by centrifugation (8,000 rpm; 10 min) and resuspended in 10 ml of MH medium (∼1 × 10^9^ CFU/ml). Fifty microliters of this cell solution was added to 48 µl of MH medium, and 2 µl of each drug from the drug library was added to wells of a white-walled 96-well microtiter plate (Corning Life Sciences, Durham, NC), achieving a concentration of 100 µM. A higher screening concentration was used in comparison to previous studies to ensure hit identification against the resilient nature of SCV (35, 36). Plates were incubated for 24 h at 37°C. Afterward 100 µl of ToxiLight AK reagent (Lonza, Basel, Switzerland) was added to each well and incubated for 30 min. Luminescence was measured using a SpectraMax M5 plate reader. The assay was performed in duplicate. Hits were identified by thresholding luminescent values normalized to the mock-treated growth control with values greater than or equal to the overall mean plus two times the standard deviation. To compensate for increased noise on certain plates, thresholding was applied independently to select plates. Additional hits were selected by removing select plates that had increased noise relative to the overall data. Secondary cell viability assays, in which ∼1 × 10^5^ CFU/ml UAMS-1112 cells were treated with each of the identified hits at 100 µM for 48 h and enumerated by plating, distinguished primary assay screening artifacts from validated hits.

### Antimicrobial susceptibility testing.

MIC testing was performed in accordance with the Clinical and Laboratory Standard Institute (CLSI) guidelines (66). In short, wells of a 96-well plate (Falcon; Corning Life Sciences, Durham, NC) were inoculated with ∼1 × 10^5^ CFU/ml of either UAMS-1112, UAMS-1, or USA300 containing serial dilutions of the indicated antimicrobial ranging from 0 to 128 µg/ml in MH media. This assay was also performed in the presence of human serum instead of MH media. The MIC was defined as the lowest concentration of antimicrobial where there was no visible bacterial growth in the wells. For UAMS-1112 and human serum, judging the MIC was difficult due to minimal growth and opaque media, respectively. Because of this, the MIC was defined as compounds that demonstrated a ≥2-log CFU/ml decrease in cell growth inhibition relative to dimethyl sulfoxide (DMSO) mock-treated cells. All MICs were confirmed by repeat testing in duplicate.

The spontaneous resistance frequencies were determined for daunorubicin, ketoconazole, rifapentine, sitafloxacin, rifampin, and gentamicin. Exponential-phase cultures of S. aureus UAMS-1, OD_600_ of 0.2, were concentrated to ∼1 × 10^9^ CFU/ml by centrifuging (13.3 × *g*; 2 min) 1 ml of culture and resuspending in 100 µl. The concentrated cell suspensions were then plated on MH agar to determine the starting inoculum, and the plates were supplemented with 2× and 4× the MIC of daunorubicin, ketoconazole, rifapentine, sitafloxacin, and rifampin. The resistance frequency was determined as the number of resistant colonies divided by the total CFU of the inoculum. Testing was performed in duplicate.

### Biofilm susceptibility assay.

Established biofilms were tested for susceptibility against validated HTS hits and gentamicin as a clinically relevant control, using minimum biofilm eradication concentration (MBEC) physiology and genetics (P&G) plates (Innovotech, Edmonton, Canada), which have previously been established with some minor modifications (67). Briefly, overnight cultures of UAMS-1 were adjusted to ∼1 × 10^6^ CFU/ml in TSB supplemented with 10% (vol/vol) pooled human plasma (Innovative Research, Novi, MI) and added to individual wells of MBEC plates at a volume of 150 µl. Plates were incubated for 24 h at 37°C with shaking at 110 rpm. After incubation, peg lids were transferred to a 96-well microtiter plate containing 200 µl of PBS and washed for 1 min with shaking at 110 rpm. Then washed peg lids were transferred to an antimicrobial challenge plate containing 2-fold serial dilutions of 200 µl of gentamicin and validated HTS drugs in TSB. The challenged biofilm was incubated for an additional 24 h at 37°C with shaking at 110 rpm. The following day, pegs were washed, transferred to a bacterial recovery plate containing 200 µl of TSB, and then sonicated at 50/60 Hz using a Bransonic 220 Ultrasonic Cleaner. Bacterial viability was immediately assessed using 100 µl from the recovery plate for enumeration and plating serial dilutions on TSB agar. Scanning electron microscopy (SEM) was also implemented to qualitatively assess the growth of the biofilm at 24 and 48 h. Growth control pegs were broken off, fixed in 2.5% glutaraldehyde/4% paraformaldehyde in 0.1 M cacodylate buffer, postfixed in 1% osmium tetroxide, dehydrated, and gold sputter coated prior to imaging. Gentamicin was compared against each of the validated hits at each tested concentration in a two-way ANOVA with Sidak’s test for multiple comparisons. Differences were considered significant for *P < *0.05. Testing was performed in triplicate.

### Cytotoxicity.

The cytotoxic effects of the validated HTS hits and gentamicin were characterized on HEK 293T cells using the XTT (2,3-bis-(2-methoxy-4-nitro-5-sulfophenyl)-5-[(phenylamino) carbonyl]-2H-tetrazolium hydroxide) cell proliferation assay (Sigma-Aldrich, St. Louis, MO). In short, HEK 293T cells maintained in Dulbecco’s minimum essential media (DMEM) supplemented with 10% fetal bovine serum (FBS), 1% penicillin-streptomycin (PEN-STR), and 1% GlutaMAX were seeded into a 96-well microtiter plate at 1 × 10^4^ cells/well and allowed to attach overnight for 18 h at 37°C in 5% CO_2_. Cells were then treated with the HTS validated hits and gentamicin diluted in DMEM without phenol red, supplemented with 10% FBS and 1% PEN-STR, and incubated for 24 h. Cells were then rinsed with PBS, and medium was replaced with DMEM without phenol red, supplemented with 10% FBS, 1% PEN-STR, and 50 µl of XTT/PMS (1:1 ratio with cell media). Cells were incubated for an additional 3 h, and the absorbance was measured at 450 nm using a BioTek Synergy Mx plate reader. Toxicity was represented as percent cell viability relative to mock-treated growth control. Assays were performed in triplicate. Gentamicin was compared against each of the validated hits at each tested concentration in a two-way ANOVA with Sidak’s test for multiple comparisons. Differences were considered significant for *P < *0.05.

## References

[1] DiMasi JA 2001 Risks in new drug development: approval success rates for investigational drugs. Clin Pharmacol Ther 69:297–307. doi:10.1067/mcp.2001.115446. 11371997

[2] DiMasi JA, Hansen RW, Grabowski HG 2003 The price of innovation: new estimates of drug development costs. J Health Econ 22:151–185. doi:10.1016/S0167-6296(02)00126-1. 12606142

[3] Wong CH, Siah KW, Lo AW 2018 Estimation of clinical trial success rates and related parameters. Biostatistics. doi:10.1093/biostatistics/kxx069. PMC6409418 29394327

[4] Ashburn TT, Thor KB 2004 Drug repositioning: identifying and developing new uses for existing drugs. Nat Rev Drug Discov 3:673. doi:10.1038/nrd1468. 15286734

[5] Barrows NJ, Campos RK, Powell ST, Prasanth KR, Schott-Lerner G, Soto-Acosta R, Galarza-Munoz G, McGrath EL, Urrabaz-Garza R, Gao J, Wu P, Menon R, Saade G, Fernandez-Salas I, Rossi SL, Vasilakis N, Routh A, Bradrick SS, Garcia-Blanco MA 2016 A screen of FDA-approved drugs for inhibitors of Zika virus infection. Cell Host Microbe 20:259–270. doi:10.1016/j.chom.2016.07.004. 27476412 PMC4993926

[6] Johansen LM, DeWald LE, Shoemaker CJ, Hoffstrom BG, Lear-Rooney CM, Stossel A, Nelson E, Delos SE, Simmons JA, Grenier JM, Pierce LT, Pajouhesh H, Lehar J, Hensley LE, Glass PJ, White JM, Olinger GG 2015 A screen of approved drugs and molecular probes identifies therapeutics with anti-Ebola virus activity. Sci Transl Med 7:290ra89. doi:10.1126/scitranslmed.aaa5597. 26041706

[7] Kaufman AC, Salazar SV, Haas LT, Yang J, Kostylev MA, Jeng AT, Robinson SA, Gunther EC, van Dyck CH, Nygaard HB, Strittmatter SM 2015 Fyn inhibition rescues established memory and synapse loss in Alzheimer mice. Ann Neurol 77:953–971. doi:10.1002/ana.24394. 25707991 PMC4447598

[8] He S, Lin B, Chu V, Hu Z, Hu X, Xiao J, Wang AQ, Schweitzer CJ, Li Q, Imamura M, Hiraga N, Southall N, Ferrer M, Zheng W, Chayama K, Marugan JJ, Liang TJ 2015 Repurposing of the antihistamine chlorcyclizine and related compounds for treatment of hepatitis C virus infection. Sci Transl Med 7:282ra49. doi:10.1126/scitranslmed.3010286. PMC6420960 25855495

[9] Feng J, Wang T, Shi W, Zhang S, Sullivan D, Auwaerter PG, Zhang Y 2014 Identification of novel activity against Borrelia burgdorferi persisters using an FDA approved drug library. Emerg Microbes Infect 3:e49. doi:10.1038/emi.2014.53. 26038747 PMC4126181

[10] Zhang L, He M, Zhang Y, Nilubol N, Shen M, Kebebew E 2012 Quantitative high-throughput drug screening identifies novel classes of drugs with anticancer activity in thyroid cancer cells: opportunities for repurposing. J Clin Endocrinol Metab 97:E319–E328. doi:10.1210/jc.2011-2671. 22170715 PMC3319218

[11] Xu M, Lee EM, Wen Z, Cheng Y, Huang W-K, Qian X, TCW J, Kouznetsova J, Ogden SC, Hammack C, Jacob F, Nguyen HN, Itkin M, Hanna C, Shinn P, Allen C, Michael SG, Simeonov A, Huang W, Christian KM, Goate A, Brennand KJ, Huang R, Xia M, Ming G-L, Zheng W, Song H, Tang H 2016 Identification of small molecule inhibitors of Zika virus infection and induced neural cell death via a drug repurposing screen. Nat Med 22:1101–1107. doi:10.1038/nm.4184. 27571349 PMC5386783

[12] Dyall J, Coleman CM, Hart BJ, Venkataraman T, Holbrook MR, Kindrachuk J, Johnson RF, Olinger GG, Jahrling PB, Laidlaw M, Johansen LM, Lear CM, Glass PJ, Hensley LE, Frieman MB 2014 Repurposing of clinically developed drugs for treatment of Middle East respiratory coronavirus infection. Antimicrob Agents Chemother 58:4885–4893. doi:10.1128/AAC.03036-14. 24841273 PMC4136000

[13] Pessetto ZY, Weir SJ, Sethi G, Broward MA, Godwin AK 2013 Drug repurposing for gastrointestinal stromal tumor. Mol Cancer Ther 12:1299–1309. doi:10.1158/1535-7163.MCT-12-0968. 23657945 PMC3707936

[14] Ventola CL 2015 The antibiotic resistance crisis: part 1: causes and threats. P T 40:277–283. 25859123 PMC4378521

[15] von Eiff C, Bettin D, Proctor RA, Rolauffs B, Lindner N, Winkelmann W, Peters G 1997 Recovery of small colony variants of Staphylococcus aureus following gentamicin bead placement for osteomyelitis. Clin Infect Dis 25:1250–1251. doi:10.1086/516962. 9402396

[16] Proctor RA, van Langevelde P, Kristjansson M, Maslow JN, Arbeit RD 1995 Persistent and relapsing infections associated with small-colony variants of Staphylococcus aureus. Clin Infect Dis 20:95–102. doi:10.1093/clinids/20.1.95. 7727677

[17] Proctor RA 2000 Microbial pathogenic factors: small-colony variants, infections associated with indwelling medical devices, 3rd ed ASM Press, Washington, DC.

[18] Tande AJ, Osmon DR, Greenwood-Quaintance KE, Mabry TM, Hanssen AD, Patel R 2014 Clinical characteristics and outcomes of prosthetic joint infection caused by small colony variant staphylococci. mBio 5:e01910-14. doi:10.1128/mBio.01910-14. 25271290 PMC4196237

[19] Kahl B, Herrmann M, Everding AS, Koch HG, Becker K, Harms E, Proctor RA, Peters G 1998 Persistent infection with small colony variant strains of Staphylococcus aureus in patients with cystic fibrosis. J Infect Dis 177:1023–1029. doi:10.1086/515238. 9534977

[20] Kahl BC, Duebbers A, Lubritz G, Haeberle J, Koch HG, Ritzerfeld B, Reilly M, Harms E, Proctor RA, Herrmann M, Peters G 2003 Population dynamics of persistent Staphylococcus aureus isolated from the airways of cystic fibrosis patients during a 6-year prospective study. J Clin Microbiol 41:4424–4427. doi:10.1128/JCM.41.9.4424-4427.2003. 12958283 PMC193812

[21] Morelli P, De Alessandri A, Manno G, Marchese A, Bassi M, Lobello R, Minicucci L, Bandettini R 2015 Characterization of Staphylococcus aureus small colony variant strains isolated from Italian patients attending a regional cystic fibrosis care centre. New Microbiol 38:235–243. 25938748

[22] Baddour LM, Christensen GD 1987 Prosthetic valve endocarditis due to small-colony staphylococcal variants. Rev Infect Dis 9:1168–1174. doi:10.1093/clinids/9.6.1168. 3423588

[23] Seifert H, Wisplinghoff H, Schnabel P, von Eiff C 2003 Small colony variants of Staphylococcus aureus and pacemaker-related infection. Emerg Infect Dis 9:1316–1318. doi:10.3201/eid0910.0302000. 14609471 PMC3033069

[24] Spanu T, Romano L, D’Inzeo T, Masucci L, Albanese A, Papacci F, Marchese E, Sanguinetti M, Fadda G 2005 Recurrent ventriculoperitoneal shunt infection caused by small-colony variants of Staphylococcus aureus. Clin Infect Dis 41:e48–e52. doi:10.1086/432577. 16080075

[25] Tuchscherr L, Kreis CA, Hoerr V, Flint L, Hachmeister M, Geraci J, Bremer-Streck S, Kiehntopf M, Medina E, Kribus M, Raschke M, Pletz M, Peters G, Löffler B 2016 Staphylococcus aureus develops increased resistance to antibiotics by forming dynamic small colony variants during chronic osteomyelitis. J Antimicrob Chemother 71:438–448. doi:10.1093/jac/dkv371. 26589581

[26] von Eiff C, Becker K, Metze D, Lubritz G, Hockmann J, Schwarz T, Peters G 2001 Intracellular persistence of Staphylococcus aureus small-colony variants within keratinocytes: a cause for antibiotic treatment failure in a patient with Darier’s disease. Clin Infect Dis 32:1643–1647. doi:10.1086/320519. 11340539

[27] Abele-Horn M, Schupfner B, Emmerling P, Waldner H, Göring H 2000 Persistent wound infection after herniotomy associated with small-colony variants of Staphylococcus aureus. Infection 28:53–54. doi:10.1007/s150100050014. 10697795

[28] Onyango LA, Hugh Dunstan R, Roberts TK, Macdonald MM, Gottfries J 2013 Phenotypic variants of staphylococci and their underlying population distributions following exposure to stress. PLoS One 8:e77614. doi:10.1371/journal.pone.0077614. 24204894 PMC3799968

[29] Lowy FD 1998 Staphylococcus aureus infections. N Engl J Med 339:520–532. doi:10.1056/NEJM199808203390806. 9709046

[30] McNamara PJ, Proctor RA 2000 Staphylococcus aureus small colony variants, electron transport and persistent infections. Int J Antimicrob Agents 14:117–122. doi:10.1016/S0924-8579(99)00170-3. 10720801

[31] Proctor RA, von Eiff C, Kahl BC, Becker K, McNamara P, Herrmann M, Peters G 2006 Small colony variants: a pathogenic form of bacteria that facilitates persistent and recurrent infections. Nat Rev Microbiol 4:295–305. doi:10.1038/nrmicro1384. 16541137

[32] Kahl BC, Becker K, Loffler B 2016 Clinical significance and pathogenesis of staphylococcal small colony variants in persistent infections. Clin Microbiol Rev 29:401–427. doi:10.1128/CMR.00069-15. 26960941 PMC4786882

[33] Sendi P, Proctor RA 2009 Staphylococcus aureus as an intracellular pathogen: the role of small colony variants. Trends Microbiol 17:54–58. doi:10.1016/j.tim.2008.11.004. 19162480

[34] Tuchscherr L, Heitmann V, Hussain M, Viemann D, Roth J, von Eiff C, Peters G, Becker K, Löffler B 2010 Staphylococcus aureus small-colony variants are adapted phenotypes for intracellular persistence. J Infect Dis 202:1031–1040. doi:10.1086/656047. 20715929

[35] Jacobs AC, Didone L, Jobson J, Sofia MK, Krysan D, Dunman PM 2013 Adenylate kinase release as a high-throughput-screening-compatible reporter of bacterial lysis for identification of antibacterial agents. Antimicrob Agents Chemother 57:26–36. doi:10.1128/AAC.01640-12. 23027196 PMC3535927

[36] Blanchard C, Brooks L, Ebsworth-Mojica K, Didione L, Wucher B, Dewhurst S, Krysan D, Dunman PM, Wozniak RAF 2016 Zinc pyrithione improves the antibacterial activity of silver sulfadiazine ointment. mSphere 1:e00194-16. doi:10.1128/mSphere.00194-16. 27642637 PMC5023846

[37] Wininger DA, Fass RJ 1996 Antibiotic-impregnated cement and beads for orthopedic infections. Antimicrob Agents Chemother 40:2675–2679. doi:10.1128/AAC.40.12.2675. 9124821 PMC163602

[38] Darouiche RO 2004 Treatment of infections associated with surgical implants. N Engl J Med 350:1422–1429. doi:10.1056/NEJMra035415. 15070792

[39] von Eiff C, Heilmann C, Proctor RA, Woltz C, Peters G, Götz F 1997 A site-directed Staphylococcus aureus hemB mutant is a small-colony variant which persists intracellularly. J Bacteriol 179:4706–4712. doi:10.1128/jb.179.15.4706-4712.1997. 9244256 PMC179315

[40] Garcia LG, Lemaire S, Kahl BC, Becker K, Proctor RA, Denis O, Tulkens PM, Van Bambeke F 2013 Antibiotic activity against small-colony variants of Staphylococcus aureus: review of in vitro, animal and clinical data. J Antimicrob Chemother 68:1455–1464. doi:10.1093/jac/dkt072. 23485724

[41] Johns BE, Purdy KJ, Tucker NP, Maddocks SE 2015 Phenotypic and genotypic characteristics of small colony variants and their role in chronic infection. Microbiol Insights 8:15–23. doi:10.4137/MBI.S25800. PMC4581789 26448688

[42] Tashiro Y, Eida H, Ishii S, Futamata H, Okabe S 2017 Generation of small colony variants in biofilms by *Escherichia coli* harboring a conjugative F plasmid. Microbes Environ 32:40–46. doi:10.1264/jsme2.ME16121. 28302951 PMC5371073

[43] Cardile AP, Sanchez CJ, Samberg ME, Romano DR, Hardy SK, Wenke JC, Murray CK, Akers KS 2014 Human plasma enhances the expression of staphylococcal microbial surface components recognizing adhesive matrix molecules promoting biofilm formation and increases antimicrobial tolerance in vitro. BMC Res Notes 7:457. doi:10.1186/1756-0500-7-457. 25034276 PMC4110374

[44] Hammer KA, Carson CF, Riley TV 2008 Frequencies of resistance to Melaleuca alternifolia (tea tree) oil and rifampicin in Staphylococcus aureus, Staphylococcus epidermidis and Enterococcus faecalis. Int J Antimicrob Agents 32:170–173. doi:10.1016/j.ijantimicag.2008.03.013. 18571379

[45] Power E 2006 Impact of antibiotic restrictions: the pharmaceutical perspective. Clin Microbiol Infect 12:25–34. doi:10.1111/j.1469-0691.2006.01528.x. 16827822

[46] Outterson K 2014 New business models for sustainable antibiotics. Chatham House (The Royal Institute of International Affairs), London, England.

[47] Projan SJ 2003 Why is big pharma getting out of antibacterial drug discovery? Curr Opin Microbiol 6:427–430. doi:10.1016/j.mib.2003.08.003. 14572532

[48] Thangamani S, Mohammad H, Younis W, Seleem MN 2015 Drug repurposing for the treatment of staphylococcal infections. Curr Pharm Des 21:2089–2100. doi:10.2174/1381612821666150310104416. 25760334 PMC8672279

[49] Jin G, Wong STC 2014 Toward better drug repositioning: prioritizing and integrating existing methods into efficient pipelines. Drug Discov Today 19:637–644. doi:10.1016/j.drudis.2013.11.005. 24239728 PMC4021005

[50] Baumert N, von Eiff C, Schaaff F, Peters G, Proctor RA, Sahl HG 2002 Physiology and antibiotic susceptibility of Staphylococcus aureus small colony variants. Microb Drug Resist 8:253–260. doi:10.1089/10766290260469507. 12523621

[51] Sadowska B, Bonar A, von Eiff C, Proctor RA, Chmiela M, Rudnicka W, Róźalska B 2002 Characteristics of Staphylococcus aureus, isolated from airways of cystic fibrosis patients, and their small colony variants. FEMS Immunol Med Microbiol 32:191–197. doi:10.1111/j.1574-695X.2002.tb00553.x. 11934563

[52] Chuard C, Vaudaux PE, Proctor RA, Lew DP 1997 Decreased susceptibility to antibiotic killing of a stable small colony variant of Staphylococcus aureus in fluid phase and on fibronectin-coated surfaces. J Antimicrob Chemother 39:603–608. doi:10.1093/jac/39.5.603. 9184359

[53] Garcia LG, Lemaire S, Kahl BC, Becker K, Proctor RA, Denis O, Tulkens PM, Van Bambeke F 2012 Pharmacodynamic evaluation of the activity of antibiotics against hemin- and menadione-dependent small-colony variants of Staphylococcus aureus in models of extracellular (broth) and intracellular (THP-1 monocytes) infections. Antimicrob Agents Chemother 56:3700–3711. doi:10.1128/AAC.00285-12. 22564838 PMC3393418

[54] Nobre LS, Todorovic S, Tavares AFN, Oldfield E, Hildebrandt P, Teixeira M, Saraiva LM 2010 Binding of azole antibiotics to Staphylococcus aureus flavohemoglobin increases intracellular oxidative stress. J Bacteriology 192:1527–1533. doi:10.1128/JB.01378-09. PMC2832537 20097858

[55] Jacobs JY, Michel J, Sacks T 1979 Bactericidal effect of combinations of antimicrobial drugs and antineoplastic antibiotics against Staphylococcus aureus. Antimicrob Agents Chemother 15:580–586. doi:10.1128/AAC.15.4.580. 464589 PMC352714

[56] Pommier Y, Leo E, Zhang H, Marchand C 2010 DNA topoisomerases and their poisoning by anticancer and antibacterial drugs. Chem Biol 17:421–433. doi:10.1016/j.chembiol.2010.04.012. 20534341 PMC7316379

[57] Akasaka T, Kurosaka S, Uchida Y, Tanaka M, Sato K, Hayakawa I 1998 Antibacterial activities and inhibitory effects of sitafloxacin (DU-6859a) and its optical isomers against type II topoisomerases. Antimicrob Agents Chemother 42:1284–1287. doi:10.1128/AAC.42.5.1284. 9593169 PMC105807

[58] Wehrli W 1983 Rifampin: mechanisms of action and resistance. Rev Infect Dis 5:S407–S411. doi:10.1093/clinids/5.Supplement_3.S407. 6356275

[59] Anderson DL 2008 Sitafloxacin hydrate for bacterial infections. Drugs Today (Barc) 44:489–501. doi:10.1358/dot.2008.44.7.1219561. 18806900

[60] Tobinick EL 2009 The value of drug repositioning in the current pharmaceutical market. Drug News Perspect 22:119–125. doi:10.1358/dnp.2009.22.2.1343228. 19330170

[61] Vergidis P, Rouse MS, Euba G, Karau MJ, Schmidt SM, Mandrekar JN, Steckelberg JM, Patel R 2011 Treatment with linezolid or vancomycin in combination with rifampin is effective in an animal model of methicillin-resistant Staphylococcus aureus foreign body osteomyelitis. Antimicrob Agents Chemother 55:1182–1186. doi:10.1128/AAC.00740-10. 21189340 PMC3067063

[62] Drancourt M, Stein A, Argenson JN, Roiron R, Groulier P, Raoult D 1997 Oral treatment of Staphylococcus spp. infected orthopaedic implants with fusidic acid or ofloxacin in combination with rifampicin. J Antimicrob Chemother 39:235–240. doi:10.1093/jac/39.2.235. 9069545

[63] Matsumoto T, Uchino K, Yamaguchi H, Yoshida S, Takahashi M, Kodama H, Hamajima S, Yonemochi R, Fujita S, Takita A, Yamanouchi N, Suzuki T, Shiozawa T, Yamaguchi F 2011 Study on the safety and efficacy of sitafloxacin—results of the use-results survey. Jpn J Antibiot 64:319–337. 22428215

[64] Proctor RA, Kahl B, von Eiff C, Vaudaux PE, Lew DP, Peters G 1998 Staphylococcal small colony variants have novel mechanisms for antibiotic resistance. Clin Infect Dis 27:S68–S74. doi:10.1086/514906. 9710673

[65] Inzana JA, Schwarz EM, Kates SL, Awad HA 2016 Biomaterials approaches to treating implant-associated osteomyelitis. Biomaterials 81:58–71. doi:10.1016/j.biomaterials.2015.12.012. 26724454 PMC4745119

[66] CLSI. 2015 Performance standards for antimicrobial susceptibility testing; twenty-fifth informational supplement. CLSI document M100-S25. CLSI, Wayne, PA.

[67] Ceri H, Olson ME, Stremick C, Read RR, Morck D, Buret A 1999 The Calgary biofilm device: new technology for rapid determination of antibiotic susceptibilities of bacterial biofilms. J Clin Microbiol 37:1771–1776. 10325322 10.1128/jcm.37.6.1771-1776.1999 PMC84946

